# Impact of Proposed Medicare Policy Changes for Botulinum Toxin Coverage on Hemifacial Spasm and Facial Dystonia

**DOI:** 10.1089/fpsam.2024.0281

**Published:** 2025-11-07

**Authors:** Alexander J. Barna, Scott J. Stephan, Priyesh N. Patel, G. Nina Lu, Linda N. Lee, Shiayin F. Yang

**Affiliations:** 1 Division of Facial Plastic and Reconstructive Surgery, Department of Otolaryngology-Head & Neck Surgery, Vanderbilt University Medical Center, Nashville, Tennessee, USA.; 2 Division of Facial Plastic and Reconstructive Surgery, Department of Otolaryngology-Head & Neck Surgery, University of Washington, Seattle, Washington, USA.; 3 Division of Facial Plastic and Reconstructive Surgery, Department of Otolaryngology-Head & Neck Surgery, Harvard Medical School, Boston, MA.

## Discussion

Hemifacial spasm and facial dystonia are hyperkinetic movement disorders that cause involuntary and abnormal movements of the facial muscles. Often the cause of these conditions is from compression or damage to the facial nerve; however, there are a variety of disorders that cause spasm of facial muscles. Although there have been advances in the management and rehabilitation of these disorders, spontaneous recovery is rare, and management of this chronic disease state is needed.^1–3^

Hemifacial spasm and facial dystonia cause significant functional impairment in multiple domains. Physically, these disorders cause difficulties with speech, the ability to eat, and vision, and also cause pain and discomfort from sustained or aberrant muscle contraction. They also greatly impact patients’ psychological and social well-being. For example, past research has shown that patients with facial movement disorders have an increased incidence of depression and anxiety symptoms that can drastically impact quality of life.^1,4,5^ This may be explained by the fact that patients have difficulty expressing themselves and become more withdrawn from social interaction. Facial expressions are a fundamental aspect of nonverbal communication, and the inability to convey emotions may lead to misinterpretation or misunderstandings in social settings that may cause patients to feel disconnected from others. Unwanted facial movements also draw attention that may lead to social stigmatization.^5^ Prior studies have found that people without facial paralysis are more likely to associate individuals with facial paralysis with negative traits, especially when individuals have the inability to perform a symmetrical dynamic smile.^2^

Botulinum toxin is the cornerstone in the treatment of various neurological disorders, particularly those involving involuntary muscle contractions, such as hemifacial spasm and facial dystonia.^1–4^ For many patients, it provides substantial relief and has been found to increase quality of life scores for patients by reducing muscle spasms and helping to restore facial symmetry.^2^ Prior studies have found that patients who receive treatment with botulinum toxin are also less likely to suffer from anxiety and depressive symptoms compared to pretreatment.^5^ Currently there is no standard treatment algorithm for hemifacial spasm or facial dystonia, and treatment plans are tailored to best meet patients’ needs. Previous literature has shown that total botulinum toxin doses range from 7.5–90 botox units (BU) for hemifacial spasm^3,6^ and from 4–220 BU across facial muscles for facial dystonia per appointment.^4^

A recent policy proposal from the CGS Administrators LLC’s Proposed Local Coverage Determination (LCD)—Botulinum Toxin Injections (DL 39857) has been written to address the off-label use of botulinum toxin injections for Medicare patients and presents various challenges as currently written.^7^ For example, one item in the proposal states that Medicare will only allow payment for one injection per site regardless of the number of injections in that site, with a “site being defined as one area.” This may be problematic, as many facial plastic surgeons treat both the affected and unaffected compensatory muscles of a patient’s face when creating an effective treatment plan, and multiple injection points are often necessary to distribute targeted doses evenly across larger muscles, ensuring a more uniform effect. In terms of botulinum toxin dosing, the policy would limit total dosage to 25–30 BU into the orbicularis oculi, procerus, mentalis, platysma, orbicularis oris, and depressor anguli oris on the side of the face affected by hemifacial spasm during an initial visit. It then states that an increase of 5–15 additional units after 1 year of treatment may be required. For many patients, 30 BU may be insufficient to achieve the desired therapeutic effects. Dong et al. found that patients who received between 33 and 66 units of botulinum toxin for treatment of hemifacial spasm had a decrease in depression and anxiety symptoms compared to pretreatment.^5^ These findings may not have been possible, and patients may not have received the full benefits of this treatment had they been limited to 25–30 BU per treatment.

Given the general nature of the policy proposal as written, it is also crucial to consider the impact on patients with nonflaccid facial paralysis (NFFP), which is characterized by aberrant nerve regeneration following injury or stress to the facial nerve. NFFP leads to unwanted facial movements that occur with voluntary facial movements, such as eye closure during speech. Development of NFFP is variable and commonly seen in approximately 10–30% of patients with long-standing facial paralysis; however, some studies report rates as high as 55.5%.^8,9^ Past research has shown that neurotoxin doses range from 2–106 BU to achieve effective symptomatic control of unwanted facial movements in these patients.^9^ Similar to those with hemifacial spasm and facial dystonia, patients who receive treatment with botulinum toxin for NFFP have improvement in overall symmetry and function of the face, have decreased burden of anxiety and depressive symptoms, and have improved quality of life scores.^10^

This policy’s most immediate concern is the impact on patient care, as there would be potential for reduced treatment efficacy for hemifacial spasm and facial dystonia. Furthermore, it is unclear how this policy may affect patients undergoing treatment with botulinum toxin for NFFP. Patients who receive inadequate dosing may result in suboptimal treatment outcomes, leaving patients with residual symptoms that can negatively impact their quality of life. Another significant concern is the potential for increased health care costs. While this policy may initially reduce costs by limiting the use of an expensive medical therapy, patients who do not achieve adequate symptom control at lower doses of botulinum toxin may require additional health care resources, such as more frequent medical visits.

This policy also heavily impacts facial plastic and reconstructive surgeons and other physicians who care for these patients, as a 30 BU cap imposes significant limitations on clinical practice. Caring for these patients is both a medicine and an art, as it requires a precise understanding of facial anatomy so that treatment plans can be tailored appropriately for the individual needs of each patient. It may also place physicians in a difficult ethical position as they would be required to adhere to Medicare guidelines yet may be aware that 25–30 BU is insufficient for their patients. Botulinum toxin is commonly manufactured in 50- or 100-unit vials that are labeled as single use per the manufacturer. Limiting the dose to 30 units would lead to excess medication waste that could instead be utilized to maximize symptomatic management for patients.

Although this policy is well intentioned, there are concerns on how it will affect patients currently undergoing treatment with botulinum toxin for various conditions that cause spasm of the facial muscles. As shown in the literature, many patients require over 30 BU to effectively manage symptoms, and limiting total neurotoxin dosage would lead to under-treatment with negative impacts on patient physical function and overall quality of life.^3,4,6,9^ By failing to account for the variability in patient needs and the complexities of treating these conditions, this policy risks compromising the quality of care for a vulnerable patient population. It is imperative that policymakers reevaluate these limitations and work with facial plastic and reconstructive surgeons and other key stakeholders to create evidence-based guidelines that meet the needs of all patients. Only by doing so can we ensure that patients with hemifacial spasm and facial dystonia continue to receive the high-quality care they deserve.
